# Metabolite Analysis of Toosendanin by an Ultra-High Performance Liquid Chromatography-Quadrupole-Time of Flight Mass Spectrometry Technique

**DOI:** 10.3390/molecules181012144

**Published:** 2013-09-30

**Authors:** Jian-Lin Wu, Elaine Lai-Han Leung, Hua Zhou, Liang Liu, Na Li

**Affiliations:** State Key Laboratory of Quality Research in Chinese Medicine, Macau Institute for Applied Research in Medicine and Health, Macau University of Science and Technology, Avenida Wai Long, Taipa postcode, Macao; E-Mails: jlwu@must.edu.mo (J.-L.W.); lhleung@must.edu.mo (E.L.L.); hzhou@must.edu.mo (H.Z.)

**Keywords:** toosendanin, metabolites, human liver microsomes, UHPLC-Q-TOF/MS, MS characterization

## Abstract

Toosendanin is the major bioactive component of *Melia toosendan* Sieb. et Zucc., which is traditionally used for treatment of abdominal pain and as an insecticide. Previous studies reported that toosendanin possesses hepatotoxicity, but the mechanism remains unknown. Its bioavailability in rats is low, which indicates the hepatotoxicity might be induced by its metabolites. In this connection, in the current study, we examined the metabolites obtained by incubating toosendanin with human live microsomes, and then six of these metabolites (M1–M6) were identified for the first time by ultra-high performance liquid chromatography-quadrupole-time of flight mass spectrometry (UHPLC-Q-TOF/MS). Further analysis on the MS spectra showed M1, M2, and M3 are oxidative products and M6 is a dehydrogenation product, while M4 and M5 are oxidative and dehydrogenation products of toosendanin. Moreover, their possible structures were deduced from the MS/MS spectral features. Quantitative analysis demonstrated that M1-M5 levels rapidly increased and reached a plateau at 30 min, while M6 rapidly reached a maximal level at 20 min and then decreased slowly afterwards. These findings have provided valuable data not only for understanding the metabolic fate of toosendanin in liver microsomes, but also for elucidating the possible molecular mechanism of its hepatotoxicity.

## 1. Introduction

**Figure 1:**
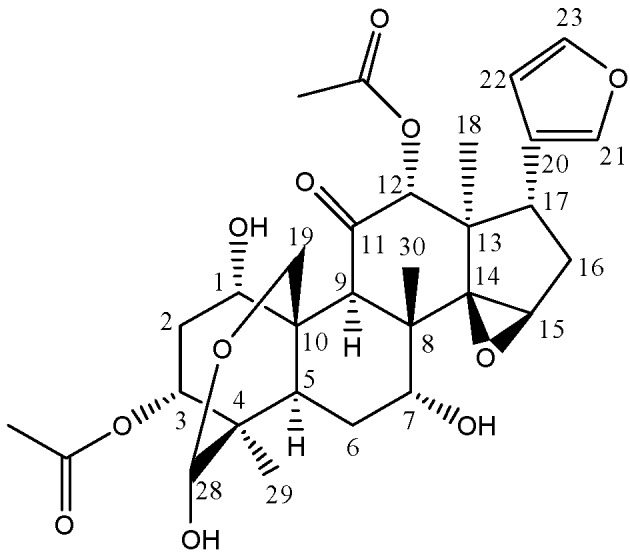
Structure of toosendanin.

Toosendanin (Figure 1) is the major active component of Toosendan fructus (the fruit of *Melia toosendan* Sieb. et Zucc.) and Meliae cortex (root bark and bark of *M. toosendan* or *M. azedarach* L.), which are usually used for the treatment of abdominal pain and as an insecticidal agent in China and other Asian countries [1]. Toosendanin-containing herbs have been used by the pharmaceutical industry for manufacturing natural products preparations [2]. A number of pharmacological reports showed that toosendanin has anti-botulism [3], growth inhibitory and apoptosis induction activities in hepatocellular carcinoma cells and other types of human cancer cells [4,5]. However, it was reported that toosendanin has significant hepatotoxicity [6,7], which seriously impairs its clinical application potential. Therefore, we have to investigate in depth toosendanin and/or its metabolites and its underlying molecular mechanisms of hepatotoxicity in order to ensure its safety in clinical usage. Such results could further expand the wide applications of toosendanin, not only based on its traditional usage, but also explore novel applications, such as development as anti-cancer agents through intensive investigations.

In respect of the mechanism of hepatotoxicity, previous studies in a rat model showed that it is associated with the production of free radical and inflammatory mediators [8], resulting in generation of reactive oxygen species (ROS), mitogen-activated protein kinases (MAPK) activation, mitochondrial dysfunction and caspase activation in primary rat hepatocytes [9]. However, whether there are toosendanin metabolites which are directly responsible for the hepatotoxicity remains unknown. Also, if present, the corresponding molecular targets need to be clarified and identified. Previous pharmacokinetic research on toosendanin in rats demonstrated that toosendanin could be quickly absorbed after oral administration and the absolute bioavailability was lower than 10% [10], which implies that the hepatotoxicity might be induced by its metabolites. However, there are no reports of *in vitro* or *in vivo* studies trying to identify its metabolites. In the current study, the *in vitro* metabolism of toosendanin was investigated by incubation of toosendanin with human liver microsomes to allow toosendanin be metabolized, and then the metabolites were efficiently measured and identified by ultra-high performance liquid chromatography-quadrupole-time of flight mass spectrometry (UHPLC-Q-TOF/MS). As a result, six metabolites have been identified, which provides valuable data for further elucidating the molecular hepatotoxic mechanism of toosendanin.

## 2. Results and Discussion

### 2.1. Mass Spectrometric Characterization of Toosendanin

**Figure 2:**
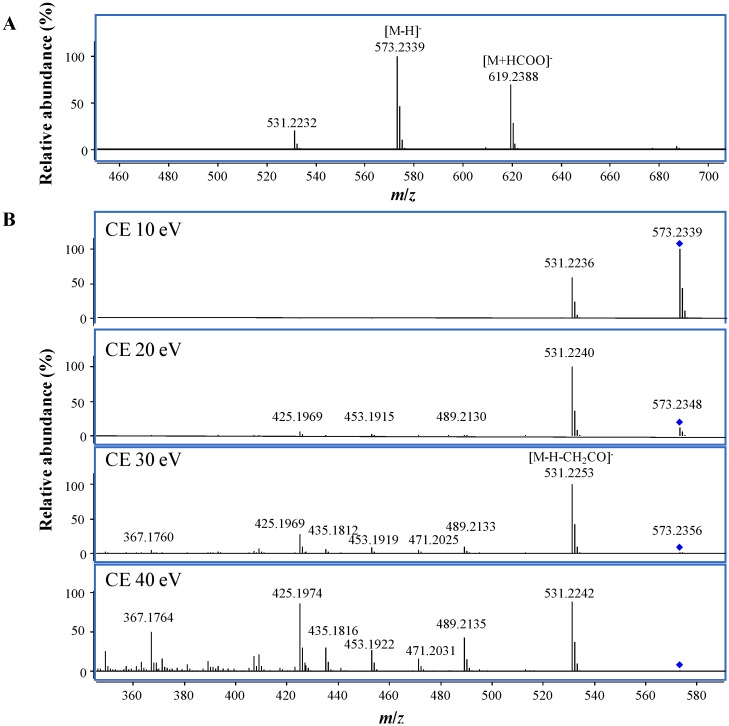
(**A**) MS and (**B**) MS/MS spectra of toosendanin at different collision cell energies (CE) in negative mode.

Q-TOF mass spectrometry can provide high resolution and accurate mass measurement of both the precursor and product ions, thus it has become more and more popular for identification of drug metabolites [11]. The molecular composition of a drug metabolite can be directly generated by Molecular Formula Generation (MFG) from the accurate masses and isotopic patterns, and the structural and substitute information can be obtained from the fragmentation ions produced by MS/MS analysis. To obtain an overall understanding of the MS fragmentation features of toosendanin can assist the identification of its metabolites. Thus, in our current study, the MS characterization of toosendanin was firstly investigated using an electrospray ion (ESI) source. Similar to previous report conducted on LCQ ion trap mass spectrometer [12], the main molecular ion was the sodium adduct ion ([M+Na]^+^) at *m*/*z* 597.2308 in positive mode (Figure S1), and the fragmentation ions at *m*/z 557.2380, 497.2170, 479.2068, 437.1958, and 419.1854 were also observed with higher abundance and identified as the fragments of [M+H−H_2_O]^+^, [M+H−H_2_O−CH_3_COOH]^+^, [M+H−2H_2_O−CH_3_COOH]^+^, [M+H−H_2_O−2CH_3_COOH]^+^, and [M+H−2H_2_O−2CH_3_COOH]^+^, respectively, from MFG, which showed in consistency with existence of two acetoxyl and two hydroxyl groups in toosendanin. In order to get more structural information on the skeleton, the MS/MS analysis was conducted using [M+Na]^+^ as the precursor due to the low abundance of [M+H]^+^. In addition to the ions produced by the neutral loss of CH_3_COOH and/or H_2_O at *m*/*z* 537.2095 ([M+Na−CH_3_COOH]^+^), 519.1996 ([M+Na−H_2_O−CH_3_COOH]^+^), and 477.1879 ([M+Na−2CH_3_COOH]^+^), the ions at *m*/*z* 491.2038 and 431.1821 were also observed and assigned to be [M+Na−H_2_O−CH_3_COOH−CO]^+^ and [M+Na−H_2_O−2CH_3_COOH−CO]^+^, which indicated the existence of ketone. In negative mode, the deprotonated ion of [M−H]^−^ was the main peak (Figure 2A), which was selected as the precursor to conduct MS/MS analysis with the collision cell energies (CE) of 10, 20, 30 and 40 eV, respectively. The results showed that the most abundant fragment was [M−H−CH_2_CO]^−^ at *m*/*z* 531.2253 (Figure 2B), and the fragments produced by the neutral loss of CH_2_CO, CH_3_COOH, H_2_O and/or CO were also observed at *m*/*z* 489.2133 ([M−H−2CH_2_CO]^−^), 471.2025 ([M−H−CH_2_CO−CH_3_COOH]^−^), 453.1919 ([M−H−CH_2_CO−CH_3_COOH−H_2_O]^−^), 435.1812 ([M−H−CH_2_CO−CH_3_COOH−2H_2_O]), and 425.1969 ([M−H−2CH_3_COOH−CO]^−^).

### 2.2. Metabolism of Toosendanin *in Vitro* by Human Liver Microsomes

In order to elucidate the possible mechanism of hepatotoxicity induced by toosendanin, the *in vitro* metabolism of toosendanin was investigated by incubating toosendanin with human liver microsomes. Equal volume of the incubation solutions were collected after incubation for 0, 10, 20, 30, 60, 90 and 120 min, respectively, and the remaining amount of toosendanin was analyzed by the newly developed UHPLC-triple quadrupole mass spectrometry (UHPLC-QQQ/MS) quantification approach, while the metabolites were identified by UHPLC-Q-TOF/MS analysis.

#### 2.2.1. Quantification of Toosendanin by UHPLC-QQQ/MS

Previously, two LC-MS analytical methods were reported to quantify toosendanin in medicinal herbs [12] and in rat plasma [10], respectively. However, due to its limitation of using single stage MS ion, [M+Na]^+^ or [M−H]^−^, for quantification, the sensitivity and dynamic linear range were unsatisfactory. Thus, in the current study a new UHPLC-QQQ/MS quantification approach was established by using MRM mode with the transition of *m*/*z* 573.2→531.2 in negative mode. A good linear regression equation, Y = 10.604X + 10.572, was obtained in the range of 10–5,000 ng/mL with the linear regression coefficient (r^2^) of 0.9997. The intra-day and inter-day variations at 20, 200, and 2,000 ng/mL were 7.1% and 7.8%, 2.8% and 4.9%, 3.4% and 5.5%, respectively. And good accuracies of 99.8, 102.2, and 99.2% were also achieved at all three concentrations been examined. The remaining amount of toosendanin at different reaction times was quantified, and the results indicated that toosendanin could be rapidly metabolized within 30 min, and hardly detected at 120 min (Figure S2), which was consistent with the pharmacokinetic results in rat [10].

#### 2.2.2. Identification of Metabolites by UHPLC-Q-TOF/MS

**Figure 3:**
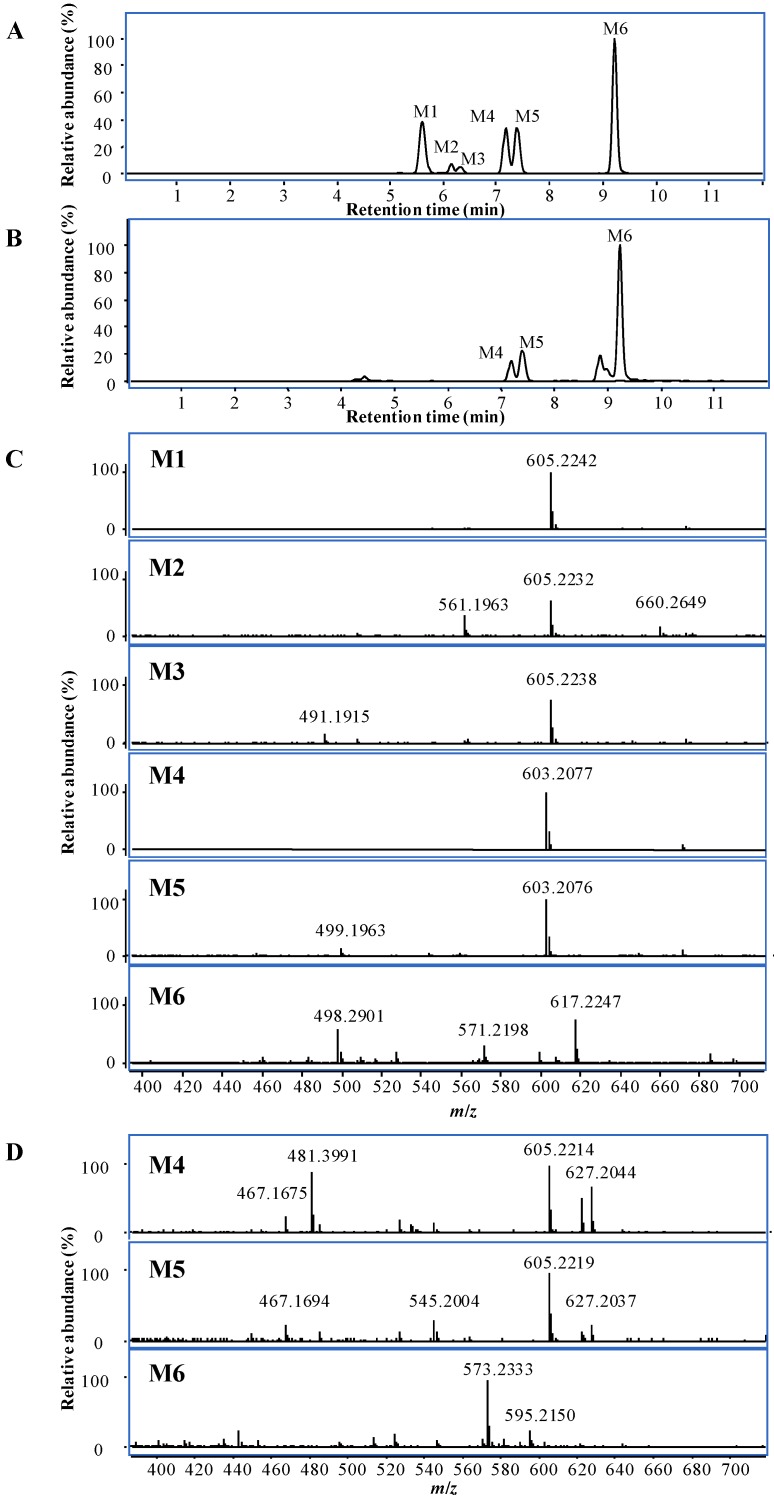
UHPLC-MS chromatogram of metabolites and their MS spectra *in vitro* at 20 min in negative mode (**A** and **C**) and positive modes (**B** and **D**).

By comparison with the sample collected at 0 min, six metabolites were detected at 5.6, 6.2, 6.3, 7.2, 7.4 and 9.2 min by UHPLC-Q-TOF/MS in negative mode (Figure 3A), while only three metabolites, M4, M5 and M6, were detected in positive mode (Figure 3B). M1, M2, and M3 had the similar *m*/*z* at 605.2242, 605.2232, and 605.2238 (Figure 3C) in negative mode, and the molecular formula of C_30_H_38_O_13_ was generated by MFG, which contains two more oxygen atoms than toosendanin.

M1 and M2 produced the similar MS/MS fragmentation features when the deprotonated ion of [M−H]^−^ was fragmented at CE of 20 eV (Figure S3A,B). The ions at *m*/*z* 563.2128, 561.2346, 545.2028, 519.2220, 501.2122, 483.2021, and 395.1858 had the higher abundance and deduced to be [M−H−CH_2_CO]^−^, [M−H−CO_2_]^−^, [M−H−CH_3_COOH]^−^, [M−H−CH_2_CO−CO_2_]^−^, [M−H−CH_3_COOH−CO_2_]^−^, [M−H−CH_3_COOH−H_2_O−CO_2_]^−^, and [M−H−CH_2_CO−CH_3_COOH−2H_2_O−CO−CO_2_]^−^, respectively, from the molecular composition generated by MFG. Different from toosendanin, most of the fragment ions with high abundance were related to the neutral loss of a CO_2_, which indicated that a carboxylic acid or lactone was produced by the oxidative metabolism.

**Scheme 1:**
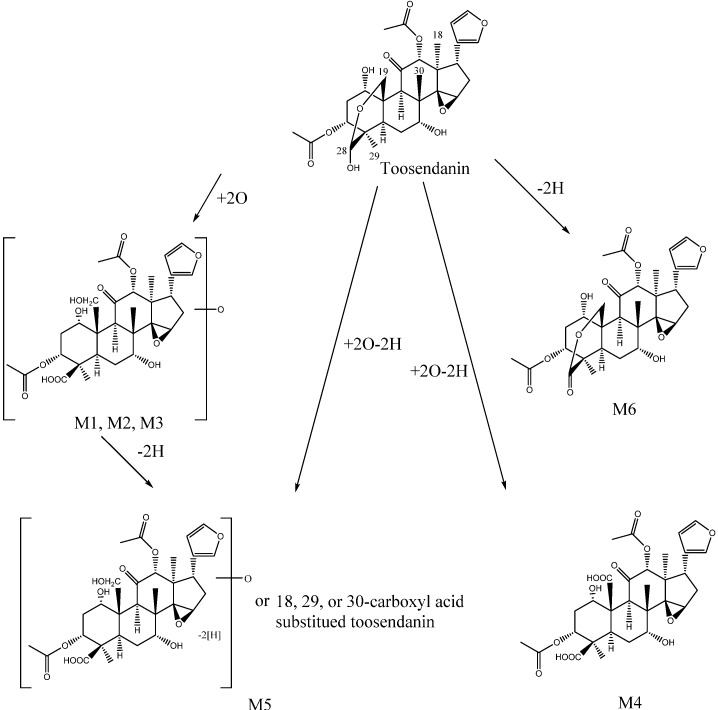
Proposed metabolic pathway of toosendanin *in vitro*.

Based on the structure of toosendanin (Figure 1), it was deduced that the most possible oxidizable position was the hemiacetal group at C-28, which could be oxidized to a carboxylic acid. Another oxygen atom might be added to the methyl, methylene, or methine groups to form a hydroxyl group (Scheme 1). At CE of 20 eV, the main fragmentation ions of M3 were [M−H−CH_2_CO]^−^ and [M−H−CH_3_COOH]^−^ at *m*/*z* 563.2149 and 545.2040, and no significant fragmentation ions indicating a loss of CO_2_ were observed (Figure S3C). When the collision cell energy was increased to 30 eV, fragment ions losing CO_2_ were detected at *m*/*z* 441.1903 ([M−H−CH_2_CO−CH_3_COOH−H_2_O−CO_2_]^−^) with high abundance (Figure S3D), in addition to the ions at *m*/*z* 519.2218 and 483.2002 which were also detected in MS/MS spectra of M1 and M2. Thus, M3 should also contain a carboxylic acid or lactone group. Besides, fragment ions losing CO were also observed at *m*/*z* 499.2004 ([M−H−CH_3_COOH−H_2_O−CO]^−^) and 457.1856 ([M−H−2CH_3_COOH−CO]^−^) with higher abundance. It was deduced that M3 was the isomer of M1 and M2, and the difference might be the hydroxylation position on the methyl, methylene, or methine groups.

The molecular formulae of M4 and M5 were determined as C_30_H_36_O_13_ by MFG from the MS spectra in both positive and negative modes (Figure 3C,D), which include two more oxygen atoms and two less hydrogen atoms than toosendanin. These two metabolites had different MS/MS fragmentation features. The base peak of M4 was *m*/*z* 395.1857 (Figure S3E), and deduced to be produced by losing CH_3_COOH, H_2_O, CH_2_CO, and two molecules of CO_2_, which indicates that two carboxylic groups formed by oxidative and dehydrogenation reactions. The existence of two carboxylic groups was further confirmed by the ion peak at *m*/*z* 455.2071, which was assigned as [M−H−CH_3_COOH−2CO_2_]^−^. In addition, the fragmentation ions losing one CO_2_ were also observed at *m*/*z* 499.1981 ([M−H−CH_3_COOH−CO_2_]^−^) and 439.1749 ([M−H−CH_2_CO−CH_3_COOH−H_2_O−CO_2_]^−^). Based on the structure of toosendanin, two carboxyl groups were proposed to be formed at C-19 and C-28 (Scheme 1). Different from M4, the main MS/MS fragmentation ions of M5 were *m*/*z* 499.1973 and 457.1864 (Figure S3F), and assigned to be [M−H−CH_3_COOH−CO_2_]^−^ and [M−H−CH_2_CO−CH_3_COOH−CO_2_]^−^. No significant ion peak losing two molecules of CO_2_ was detected, which showed only one carboxylic group being produced in M5. Based on the common oxidative metabolic pathway of phase I enzyme [13], the carboxylic acid was deduced to be formed from the methyl group at C-18, C-29, or C-30 by the oxidative reaction. Moreover, there is also a possibility that M5 possesses a carboxylic acid at C-28 and an additional aldehyde or ketone formed by the dehydrogenation of an alcohol from M1, M2 or M3 (Scheme 1).

The molecular formula C_30_H_36_O_11_ was generated by MFG for M6, and it was identified as a dehydrogenation product of toosendanin. Different from other metabolites, the main peak of M6 in MS spectrum in negative mode was the adduct ion of [M+HCOO]^−^ at *m*/*z* 617.2247 (Figure 3C), while the ion of [M−H]^−^ at *m*/*z* 571.2198 had low abundance. Thus, the target MS/MS analysis was conducted using [M+HCOO]^−^ as a precursor ion. The MS/MS fragmentation ions with high abundance were observed at *m*/*z* 527.2274, 467.2100, 425.1953, and 407.1842 (Figure S3G), which were assigned as the fragmentation ions of [M+HCOO−HCOOH−CO_2_]^−^, [M+HCOO−HCOOH−CH_3_COOH−CO_2_]^−^, [M+HCOO−HCOOH−CH_3_COOH−CH_2_CO−CO_2_]^−^ and [M+HCOO−HCOOH−CH_3_COOH−CH_2_CO−H_2_O−CO_2_]^−^, and all of them were related to the neutral loss of one CO_2_ molecule, indicating the existence of a carboxylic acid group or lactone. Based on the oxidative metabolic pathway of phase I enzyme [13] and the structure of toosendanin, it was deduced that a lactone was formed at C-28 by the dehydrogenation (Scheme 1).

The relationship between the amounts of metabolites and reaction times was shown in Figure S2. The results indicated that the amounts of M1, M2, M3, M4, and M5 rapidly increased along with the reaction time and reached plateau at 30 min, while the amount of M6 reached maximal at 20 min and then slowly decreased. Besides, there is a furan ring in toosendanin, and some previous reports showed that it could be easily metabolized, however, *cis*-enedione or *cis*-enedial metabolites are unstable and can be quickly further conjugated with biomolecules, such as proteins or DNA, or small nucleophilic agents like glutathione [14,15]. In our current study, only supernatants of the incubation solutions were investigated and no additional nucleophilic agent was added, thus, the metabolism of furan was not considered during the identification of metabolites. However, the covalent modification of furan metabolites to the proteins might induce the hepatotoxicity [16]. As such, the metabolism of furan ring in toosendanin as well as the relationship between its metabolites and the hepatotoxic mechanisms needs to be further investigated.

## 3. Experimental

### 3.1. Chemicals and Reagents

Toosendanin with a purity of >98% was bought from National Institutes for Food and Drug Control (Beijing, China). Human pooled liver microsomes and NADPH-generating solutions A and B were provided by BD Gentest (San Jose, CA, USA). Acetonitrile (HPLC grade) was purchased from Anaqua Chemicals Supply Inc. Limited (Houston, TX, USA). Water was made by a Milli-Q Ultrapure water system with the water outlet operating at 18.2 MΩ (Millipore, Billerica, MA, USA).

### 3.2. Incubation of Toosendanin *In Vitro* with Human Liver Microsomes

Toosendanin (5 μg/mL) was added into potassium phosphate buffer (pH 7.4; 0.1 M) and then mixed with NADPH-generating solutions A and B, and pre-incubated at 37°C for 5 min. Human pooled liver microsomes (1 mg/mL) was added to start the metabolic reaction. Equal volume of incubation solutions were collected at 0, 10, 20, 30, 60, 90 and 120 min, respectively, and mixed with 5 volumes of iced methanol and put on the ice to quench the reaction. After centrifugation at 15,000 *g* for 10 min, the supernatants were collected and dried at 40 °C by CentriVap Vacuum Concentrator (Labconco, Kansas City, MO, USA). The residues were reconstituted in 50% methanol and centrifuged prior to UHPLC-MS analysis. The control samples without toosendanin were also prepared in parallel for each time point.

### 3.3. Quantitative Analysis of Toosendanin by UHPLC-QQQ/MS

An Agilent 1290 infinity UHPLC system (Agilent Technologies, Santa Clara, CA, USA) with binary pump, auto-sampler, thermostatted column compartment coupled with 6460 QQQ/MS system was used for the quantification of toosendanin. An Agilent UHPLC Eclipse Plus C18 column (2.1 × 50 mm, 1.8 μm) was used with a flow rate of 0.35 mL/min. The mobile phase consisted of water containing 0.1% formic acid (A) and acetonitrile containing 0.1% formic acid (B) with the following gradient: 0–0.2 min, 5% B; 0.2–6 min, the linear gradient from 5% to 55% B; 6–6.5 min, the linear gradient from 55% to 95% B; 6.5–6.9 min, 95% B; 6.9–7 min, return to 5% B. The injection volume was 2 μL. Mass spectrometry was performed in negative ESI mode, and the parameters were set as follows, dry gas temperature at 325 °C, dry gas flow at 10 L/min, the nebulizer at 40 psi, and the capillary voltage at 4000 V. Multiple reaction monitoring (MRM) with the transition from *m*/*z* 573.2 to 531.2 was applied, and the fragmentor and collision cell energy were set at 192 V and 16 eV, respectively. MS data were collected in centroid mode over a range of *m*/*z* 100–1,000. 

Toosendanin stock solution (1 mg/mL) was dissolved in methanol, and then serially diluted with 50% methanol to provide the working solutions from 10 to 5000 ng/mL. The calibration curve was established by plotting peak area *vs.* the concentrations. For the method validation, samples spiked with known quantities of toosendanin at low, medium and high concentrations corresponding to the calibration curve were prepared and analyzed in parallel. Intra- and inter-day variations expressed as relative standard deviation (RSD) were used to determine the precision of the measurement, while the accuracy was calculated by dividing the concentration measured by the spiked concentration.

### 3.4. Qualitative Analysis by UHPLC-Q-TOF/MS

An Agilent 1290 infinity UHPLC with binary pump, auto-sampler, thermostatted column compartment coupled with 6550 Q-TOF/MS system was used for the study on MS characterization and metabolites of toosendanin. The mobile phase consisted of water containing 0.1% formic acid (A) and acetonitrile containing 0.1% formic acid (B) with the following gradient: 0–0.5 min, 5% B; 0.5–8 min, linearly increased B from 5% to 30%; 8–9 min, linearly increased B from 30% to 95%; 9–10.5 min, 95% B; 10.5–10.6 min, returned back to 5% B. The injection volume was 2 μL. Dual Agilent Jet Stream Electrospray ion source (dual AJS ESI) was used and run at both positive and negative modes. The temperatures of dry gas and sheath gas were set at 250 and 300°C, while the flow rates were 15 and 11 L/min, respectively. The nebulizer was 25 psi, and the capillary and nozzle voltage were set at 4,000 and 500 V for positive mode and 4,000 and 1,500 V for negative mode. For MS/MS analysis, targeted MS/MS mode was selected, and the collision cell energy was set at 10, 20, 30 and/or 40 eV.

## 4. Conclusions

The *in vitro* metabolism of toosendanin was studied by UHPLC-MS analysis. It was found that toosendanin could be quickly metabolized by human liver microsomes and six metabolites were identified for the first time. Among these metabolites, M1-M5 were stable for at least 120 min, while the amount of detected M6 reached a maximum at 20 min and then decreased afterwards. These findings provide valuable data for elucidating the metabolic fate of toosendanin, which may support further intensive investigations for uncovering the underlying molecular mechanisms of its hepatotoxicity.

## Supplementary Materials

Supplementary materials can be accessed at: http://www.mdpi.com/1420-3049/18/10/12144/s1.
