# Supplementary material for: Metabolite Analysis of Toosendanin by an Ultra-High Performance Liquid Chromatography-Quadrupole-Time of Flight Mass Spectrometry Technique
Source: Molecules. 2013 Sep 30;18(10):12144–53. doi: 10.3390/molecules181012144 (PMC6270517; doi:10.3390/molecules181012144)

Supplementary Materials

Figure S1. MS (**A**) and MS/MS spectra (**B**) of toosendanin in positive mode.

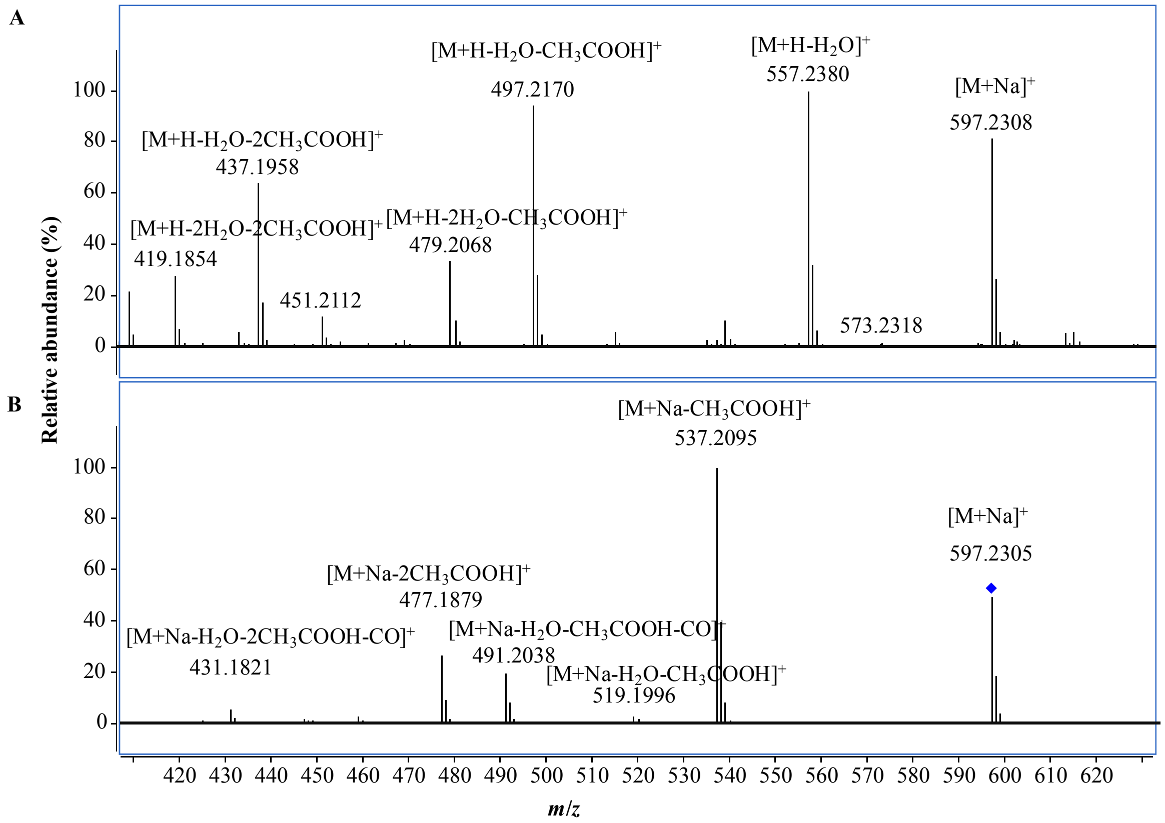

Figure S2. Amount-time relationship of toosendanin (TSN) and its six metabolites *in vitro*.

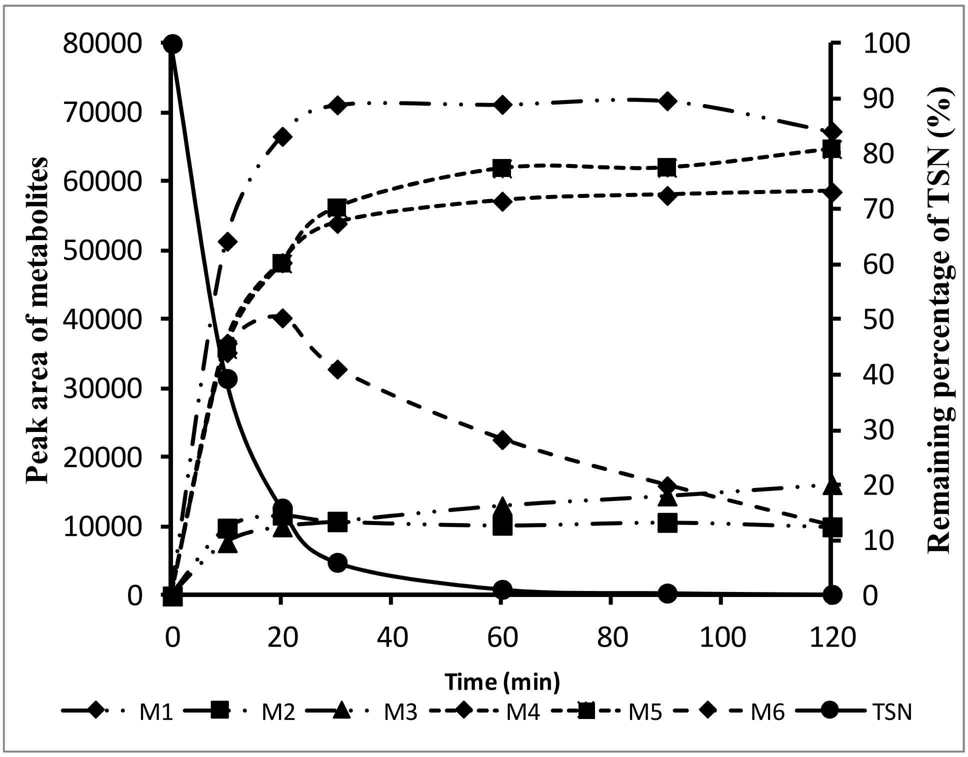

Figure S3. MS/MS spectra of metabolites *in vitro*, M1 (**A**), M2 (**B**), M3 (**C**), M4 (**E**), M5 (**F**), and M6 (**G**) at CE of 20 eV and M3 at CE of 30 eV (**D**).

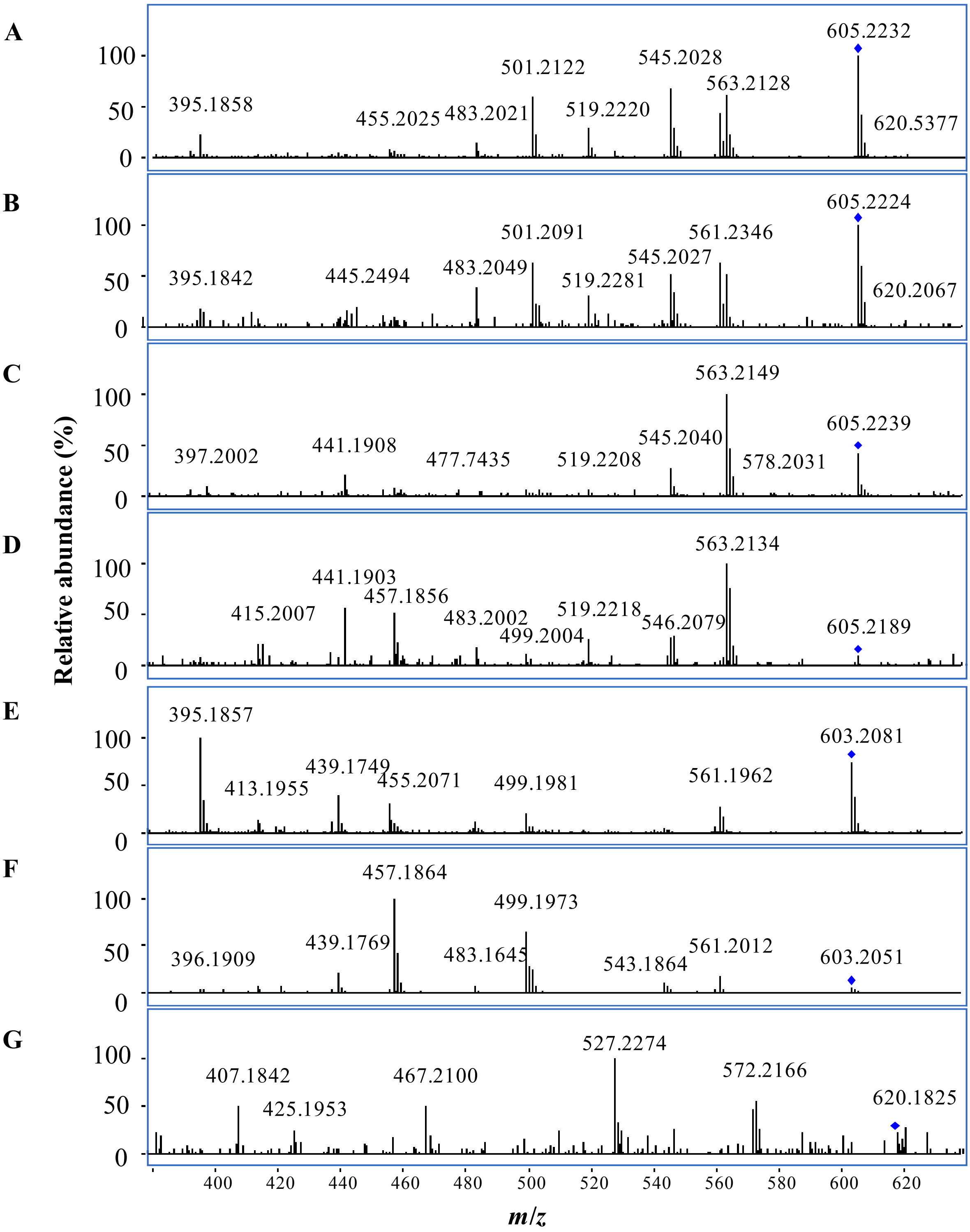

Supplement: Supplementary file 1 [file molecules-18-12144-s001.pdf]
